# Plate Waste Forecasting Using the Monte Carlo Method for Effective Decision Making in Latvian Schools

**DOI:** 10.3390/nu14030587

**Published:** 2022-01-28

**Authors:** Sergejs Kodors, Anda Zvaigzne, Lienite Litavniece, Jelena Lonska, Inese Silicka, Inta Kotane, Juta Deksne

**Affiliations:** 1 Institute of Engineering, Faculty of Engineering, Rezekne Academy of Technologies, 115 Atbrivosanas Aleja, LV-4601 Rezekne, Latvia; 2 Research Institute for Business and Social Processes, Faculty of Economics and Management, Rezekne Academy of Technologies, 115 Atbrivosanas Aleja, LV-4601 Rezekne, Latvia; Anda.Zvaigzne@rta.lv (A.Z.); Lienite.Litavniece@rta.lv (L.L.); Jelena.Lonska@rta.lv (J.L.); Inese.Silicka@rta.lv (I.S.); Inta.Kotane@rta.lv (I.K.); jd07003@edu.rta.lv (J.D.)

**Keywords:** applied computing, catering services, food loss, leftovers, modeling and simulation, plate waste, optimization, sustainability

## Abstract

Food waste is a global problem, which becomes apparent at various stages of the food supply chain. The present research study focuses on the optimization of food consumption in schools and effective food management through data-driven decision making within the trends: zero food waste and digital transformation. The paper presents a plate waste forecasting system based on mathematical modeling and simulation using the Monte Carlo method, which showed an RMSE equal to ±3% and a MAPE of 10.15%. The solution based on the simulator provides a possibility to better understand the relationship between the parameters investigated through data visualization and apply this knowledge to train managers to make decisions that are more effective. The developed system has multi-disciplinary application: forecasting, education and decision making targeted to reduce food waste and improve public health and food management in schools.

## 1. Introduction

According to the Food and Agriculture Organization of the United Nations (FAO), one-third of the world’s food output—approximately 1.3 billion tons a year—is discarded [1]. Food waste and its reduction is an increasingly important problem for local, national and European policymakers, as well as for international organizations and researchers of various fields [2]. Some researchers such as Cattaneoa, Sánchez, Torero and Vos (2021) [3] emphasize that, despite the current public policies analyzed in a number of research studies in various countries aimed at reducing food waste and wastage, there were still gaps in information, e.g., regarding how food waste is measured and monitored, gains from reducing food waste, etc. Owing to a number of research studies by Thyberg and Tonjes [4,5], the problem of food waste and the reduction in its impact gains attention among the public and policymakers.

Research shows that large amounts of food waste are generated during the consumption phase, which includes both eating meals out and at home [6]. Households mostly consume food at home, while the catering sector represents households eating meals out. The catering services sector includes both non-commercial and commercial establishments [7], e.g., restaurants, educational institution canteens, etc. Researchers Yui and Biltekoff [8] emphasize that food waste is a significant problem at educational institutions. It should be noted; however, that school canteens are a controlled environment where educational campaigns could be conducted to reduce food waste [9]. Schools can help to improve educatees’ eating habits by educating them about nutrition and raising their awareness of the role of food [10].

Despite the growing public, academic and government attention to the problem of food waste, there are still little information and few research studies on the amounts of food waste generated by school canteens, as well as the possibilities of measuring and reducing the food waste, e.g., in Latvia.

To achieve the minimization of plate waste in schools, it is important to develop a digital decision-making tool for effective food management in the schools, which will be based on three pillars: (1) knowledge and education; (2) monitoring and forecasting; (3) data updating and precise decision making.

Intervening in food-service operations to conduct field experiments and build preventive strategies is costly and not always possible [11], which is reinforced due to COVID-19 nowadays. The EU Horizon 2020 project “REFRESH” emphasizes the need to model the generation of food waste at both the consumer and producer levels [12].

The aim of the research is to develop a mathematical model and a simulator, which can be applied to forecast plate waste and can be integrated into a decision-making system for effective food management in schools.

The case study examined the plate waste generated in the secondary schools of Rezekne city in Latvia. There was restricted access to the schools due to COVID-19 during the study; therefore, it was problematic to collect enough data to apply the data-based forecasting methods. As a result, it was decided to develop a decision-making tool for effective food management based on the feature-based forecasting method. The Monte Carlo method was a good choice because scientific databases contained studies about various parameters related to a school meal, as well as some statistical data could be collected using an anonymous survey or expert knowledge.

## 2. Related Work

The simulation approach is mostly applied to investigate the global strategies related to food supply chain organization, policies and other logistic problems by using an agent-based model. For example, Craven and Krejci [13] developed a simulator to investigate producer disintermediation from a food hub in the regional food supply system. This simulator was developed with consideration to be a decision support tool for food hub managers; however, Van Voorn et al. [14] assessed efficiency and resilience in stylized food supply chains by using the simulation approach. Scalco et al. [15] applied the agent-based model to meat consumption by the population in Britain to investigate various marketing campaigns and price impacts on meat consumption. Nowadays, many research studies focus on the COVID-19 pandemic in relation to food supply chains. For example, Achmad et al. [16] studied robust food supply chain strategies by determining the optimum food hub location and food network to maintain food security, which is robust against disruptions and uncertainties during the COVID-19 pandemic.

Considering the plate waste subject, Ravandi and Jovanovic [17] applied the agent-based model to capture the dynamics between plate waste, food surplus and the facility organization setup. They measured the impact of plate size on food waste, indicating the need for optimizing food preparation along with designing choice environments that encourage guests to avoid taking more food than they need. The plate waste is more studied through other approaches, for example, the modeling approach. Dolnicar and Juvan [18] developed a model of “Drivers of plate waste” based on an interview with hotel staff. This model was extended and supported with statistical data later [19]. In addition, the authors investigated a relationship of plate waste with some impact factors by using statistical analysis, as was conducted by Anderson et al. [20], who researched a portion plate impact on food waste.

The primary objective of our research study is related to the prediction of food waste in schools. Traditionally, data-based methods are applied to make predictions, as was studied by Malefors et al. [21], who experimented with different forecasting models to predict guest attendance in school catering. As a result, they achieved an average percentage error of 2–3%. Lately, Malefors et al. [22] completed similar research on guest prediction during the COVID-19 pandemic; however, it was impossible to collect sufficient statistical data due to the restrictions related to COVID-19; therefore, it was required to find some feature-based solution, and the approach with modeling and simulation was an effective decision to overcome a problem with restricted data because scientific databases provided quantitative data and information on impact factors and eating models. Several comprehensive studies are provided by Fagerberg et al. [23], Martins et al. [24] and Zandian et al. [25]. Additionally, a simulation approach is a white-box solution, which provides the possibility to explain a relationship between parameters, while a simulator can be applied for decision making and education that opens multi-directional application for the presented solution, considering the target group—the school administration.

## 3. Materials and Methods

### 3.1. Methodology and Research Ethic

The Monte Carlo model was developed using the synergy of many different methods. Statistical data for the simulator were collected using the following methods: (1) an anonymous survey of parents; (2) literature review; (3) observations of schools. The business logic of the simulator was developed using the following methods: prototyping and experimental development. The first version of the prototype was presented at an international scientific conference and contained a minimal set of properties, which was based on expert assumptions [26]. This simulation model was extended with new parameters and based on statistical data.

To collect statistical data for the simulator, an anonymous survey of parents was conducted from 7 June 2021 to 7 July 2021. The questionnaire obtained ethical approval from the Scientific Council of Rezekne Academy of Technologies on 18 May 2021 (No. 16.1/12). All the participants were fully informed that anonymity is assured, why the research is being conducted, how their data will be used and about any associated risks. Only 24 responses were received back, yet it was a sufficient number to develop a conceptual model for the simulator and test it.

**Figure 1:**
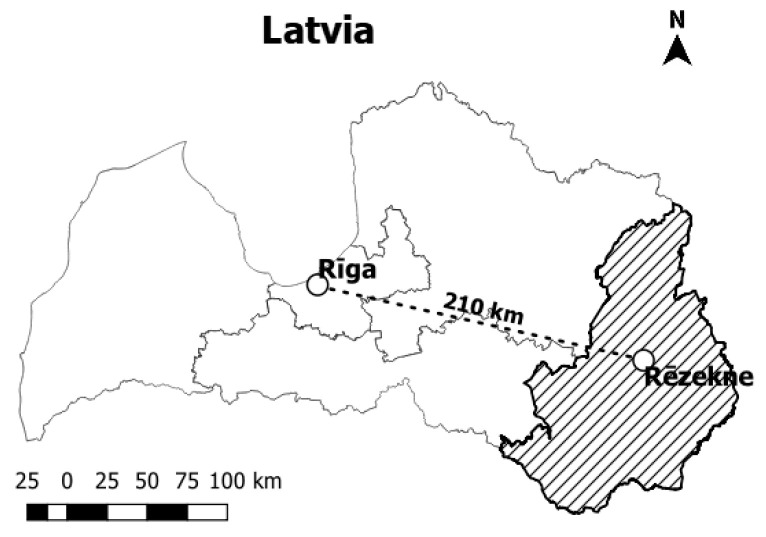
Map with the observation location.

To develop the simulation model, a field study was conducted in schools of Rezekne city, Latgale region of the Republic of Latvia. Rezekne is the seventh-largest city in the Republic of Latvia and the largest eastern border city of the European Union (Figure 1). The field study was conducted in seven secondary schools of Rezekne. During the observations, the emphasis was put on quantitative measurements of uneaten food (plate waste) by examining at least 7000 school lunch plates. To measure food intake duration and path time, not influencing children and excluding them from observations, the FIFO (first in, first out) method was applied, recording only the time when the first and last child entered and left the canteen.

The business logic of the Monte Carlo model was described using a flowchart and pseudocodes (see Section 3.3) based on the experimental development of the simulator by using *Python* and *Jupyter Notebook*.

### 3.2. Important Parameters

Various parameters were investigated to improve our Monte Carlo model. Each parameter is described in a separate subchapter: (1) a portion size (Section 3.2.1); (2) an eating rate (Section 3.2.2); (3) eating habits (Section 3.2.3); (4) children’s preferences (Section 3.2.4).

#### 3.2.1. A portion Size

**Figure 2:**
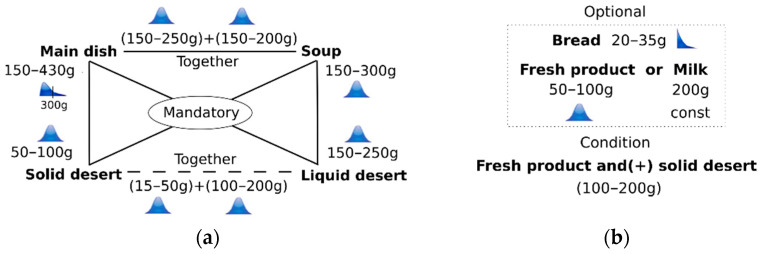
Generation model of a lunch portion: (**a**) mandatory part; (**b**) optional part.

The nutritional norms for Latvian schools are defined in regulation *No. 172 “Regulations Regarding Nutritional Norms for Educatees of Educational Institutions, Clients of Social Care and Social Rehabilitation Institutions and Patients of Medical Treatment Institutions*”. According to the version of 28 August 2020, which was in force when the study was completed, three types of menu had to be provided at school: (1) the main dish and soup (MS); (2) the main dish and dessert (MD); (3) or soup and dessert (SD). The main nutritional norms for educatees of general primary and secondary education institutions are defined by the following parameters (using minimal and maximal values): energy value (kcal), proteins (g), fats (g) and carbohydrates (g); which are grouped by grades 1–4, 5–9 and 10–12; however, the quality of nutrition is defined by many parameters, e.g., another food product must be included in the meal each day: (1) food products rich in complex carbohydrates; (2) vegetables, fruit or berries (including in fresh form); (3) food products rich in proteins; (4) milk or dairy or cultured products. At the same time, the weekly menu must contain: (1) at least 200 g (net) of lean meat or fish (fillet); (2) at least 225 g (net) of potatoes; (3) at least 450 g of dairy, cultured products or products rich in milk proteins (cottage cheese, cheese); (4) at least 700 g (net) of vegetables (except for potatoes) and fruit or berries, of which at least 250 g in fresh form. Further, the legislation defines many other requirements and restrictions, which must be considered when developing a menu. The integration of all these requirements into the simulator is a challenging task; therefore, the review of online published menus was completed to simplify a portion generation model by developing a special classification system applicable to the simulator. The resulting model is depicted in Figure 2. The portion is expressed by Equation (1), but their probability distributions are depicted in Table 1.

(1) p=m+s+sd+ld+b+fp+mk

where *p*—a portion size (g), *m*—a main dish (g), *s*—a soup (g), *sd*—a solid dessert (g), *ld*—a liquid dessert (g) such as a juice or a tea with sugar, *b*—bread (g), *fp*—a fresh product (g), *mk*—milk (g).

Additionally, the review showed that each type of menu has its own probability: (a) the main dish and a soup—65%; (b) the main dish and a dessert—30%; (c) a soup and a dessert—5%.

Fruits belong to categories “solid dessert” (*Sd*) or “fresh products” (*Fp*). This relation was described as a logical condition *AND*. The review of the menu showed that a combination of fresh products and solid desserts belongs to the range of 100–200 g. The content of lunch portions, which were monitored during the observation time, are depicted in Table 2 considering Equation (1).

#### 3.2.2. An Eating Rate

A literature analysis identified a paper by Zandian et al. [25], which provides measurements of a school lunch eating rate. They modeled cumulative food mass intake using Equation (2):

(2) $$e\left(t\right)=kt^{2}+v_{0}t,$$

where *k*—is the degree of eating rate acceleration over the course of the meal, and the $v_{0}$—is the initial speed of eating. According to Zandian et al. [25] measurements, girls had the initial eating rate equal to 34(5) g/min and the deceleration −2.6(1.4) g/min^2^, while boys −42(7) g/min and −2.7(1.1) g/min^2^, respectively; however, considering the integration of the food mass intake function (see Equation (3)), the declared deceleration must be doubled.

(3) $$e\left(t\right)=\int v\left(t\right)dt=\int \left(v_{0}+at\right)dt=v_{0}t+\frac{at^{2}}{2}+C.$$

As a result, the eating rate deceleration of girls was −5.2(2.8) g/min^2^ and −5.4(2.2) g/min^2^ for boys. Finding the extremum time (Equation (4)), it is possible to find the maximal food mass intake (Equation (3)) until the eating rate achieves zero.

(4) $$v_{0}+at=0.$$

Using the average initial speed and the average deceleration, the maximal intake must be 111.15 (g) within 6.5 (min) for girls and 163.3 (g) within 7.7 (min) for boys. That is strongly smaller than the declared average food intake and lunch duration: (a) girls—258(38) g and 10.7(2.1) min; (b) boys—289(73) g and 8.8(1.8) min.

**Figure 3:**
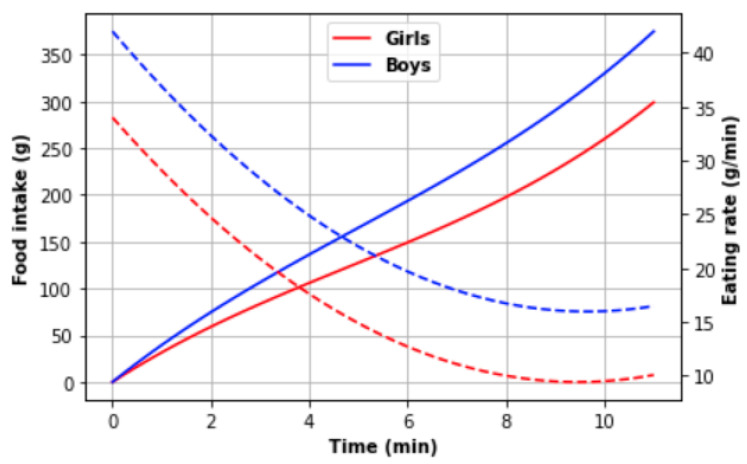
Combined diagram: solid line—food mass intake amount, dashed line—eating rate.

According to the conclusions of experts, children are eating at a decelerating speed in the first part of a lunch, then maintain a constant speed of eating [26]; therefore, the food intake model with eating speed deceleration could be applied to correct the previous calculation differences (see Equation (5)):

(5) $$e\left(t\right)=\int v\left(t\right)dt=\int \left(v_{0}+a\left(t\right)t\right)dt=\int \left(v_{0}+a_{0}t+xt^{2}\right)dt=v_{0}t+\frac{a_{0}t^{2}}{2}+\frac{xt^{3}}{3}+C$$

where $a_{0}$—is the initial eating speed acceleration, but *x*—the eating speed acceleration. Recalculation provided the following values: (a) girls—0.55 g/min^3^; (b) boys—0.56 g/min^3^. This new model is depicted in Figure 3.

However, Fagerberg et al. [23] applied a more simple model for food intake speed calculation—constant average speed (see Equation (6)):

(6) $$\bar{v}=\frac{E}{t}$$

where *E*—food mass intake during time *t*.

The research study by Fagerberg et al. [23] has shown that there is a strong correlation (Pearson’s *r* = 0.667, *p* < 0.001) between the objective eating rate (g/min) and food mass intake (g) during school lunch. Additionally, the correlation between the BMI z-score and eating speed: “a BMI z-score below 0 had a significantly lower eating rate (g/min) vs. students who had a BMI z-score equal to or above 0 (28.2 g/min vs. 35.9 g/min, respectively), the mean difference = −7.7 g/min, *p* = 0.012, 95% CI −14 g/min to −1.8 g/min” [23]; however, “slow eaters” have smaller food mass intake than “fast eaters”. Comparing the eating speed between Swedish and Greek students, a significant difference was not found. The descriptive statistics of the mentioned study were presented as a scatterplot. The minimal speed was near 10 g/min and the maximal—95 g/min (in the case of the 1st observation). The linear function is approximately equal to Equation (7):

(7) E(v)=6.8v+125

Therefore, the average speed depending on food mass intake can be found by Equation (8):

(8) $$v\left(E\right)=\frac{E-125}{6.8}$$

It is possible to find the average speed of the model proposed by Zandian et al. [25] using Equation (9):

(9) $$v_{0}t+\frac{a_{0}t^{2}}{2}+\frac{xt^{3}}{3}=\bar{v}t,$$

the results are as follows: (a) the average eating speed of girls is 27.16 g/min; (b) boys—32.7 g/min, which are comparable with the values mentioned by Fagerberg et al. [23].

Zandian et al. [25] and Fagerberg et al. [23] conducted their study on the main dish. A soup is a liquid substance; therefore, the eating rate of soup must be higher. Ferriday et al. [27] proposed a study on tomato soup (400 mL) consumed through tubing: (a) slow rate—30 mL/min; (b) fast rate—118 mL/min; in other words, approximately 30 g/min and 118 g/min. Traditionally, a soup contains some solid mass; the normal rate can be a doubled eating rate of the main dish.

To measure food intake duration and path time, not influencing children and excluding them from observations, the FIFO (first in, first out) method was applied, recording only the time when the first and last child entered ($T=\{t_{min};t_{max}\}$) and left the canteen ($T\prime =\{t_{min}^{\prime };t_{max}^{\prime }\}$). The path time was calculated as a mean of means ($\mu_{\bar{T}}$ and $\mu_{{\bar{T}}^{\prime }}$ respectively); however, the food intake duration was calculated as a mean of means ($\mu_{{\bar{T}}^{\prime }-\bar{T}}$) after subtracting the entering time from the leaving time. 

The observations at the schools showed the average lunch consumption time was equal to 8.1 min. It must be considered that children did not eat the whole portion, and plate waste was produced; however, the average eating rate was 47 g/min.

Considering the portion mass without drinking (390–540 g) and Equation (7), the eating rate must belong to the range [39; 61]. Additionally, Zandian et al. [25] mention that boys and girls eat faster in restricted time, 166% and 183%, respectively; therefore, the observed eating rate satisfies the ranges investigated by other authors.

Therefore, the eating rate of 35(5) g/min and a linear model can be a compromise for the simulator because the ideal conditions cannot always be provided, and children can feel some internal or external stress; however, the model with a decreasing speed (Equation (5)) can obtain the characteristics of a linear model for a restricted lunch time frame ($a_{0}\to 0$ and x→0).

#### 3.2.3. Children’s Eating Habits

**Figure 4:**
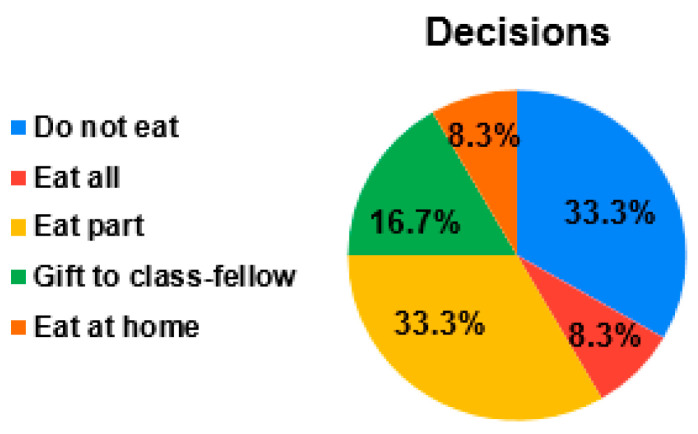
Children’s decisions concerning rejected food.

Food preferences are the product of an interplay between genetic and environmental factors that result in substantial individual differences to the extent to which children are suspicious and fussy about food in general and in their likes and dislikes for specific foods [28]. De Cosmi et al. [29] provided a comprehensive review of children’s eating and activity behaviors influenced by both intrinsic (genetics, age, gender) and extrinsic (family, peers, community, and society) factors. Eating habits influence children’s decision making; therefore, the objectives of our survey were to identify children’s decisions related to hated food and their frequency (see Figure 4), as well as to investigate how children meet their satiation and the competitive food impact on school lunches (see Table 3 and Table 4). The competitive food probability was calculated, adding up the answers of subcategories. Somewhere 30% of children eat competitive food; however, 67% of children decide to stop eating if time is not sufficient to return to a classroom.

#### 3.2.4. Children’s Food Preferences

Children from different socioeconomic regions have different food preferences [30], e.g., Ragelienė [31] discussed the differences between the food preferences of Lithuanian and Danish children. It means that local data must be applied for the simulation.

Our survey investigated three categories of food: (1) soup; (2) the main dish; (3) sweet drinks (see Table 5).

If a whole list of food products and recipes is applied for a survey to investigate children’s preferences, it is a time-consuming process; children also cannot have gastronomic experience or cannot know their titles. Additionally, these preferences can be impacted and modeled; however, a restricted list minimizes the school freedom related to menu development; therefore, it was decided to leave only one parameter for input—the number of children unsatisfied with the school meal.

**Figure 5:**
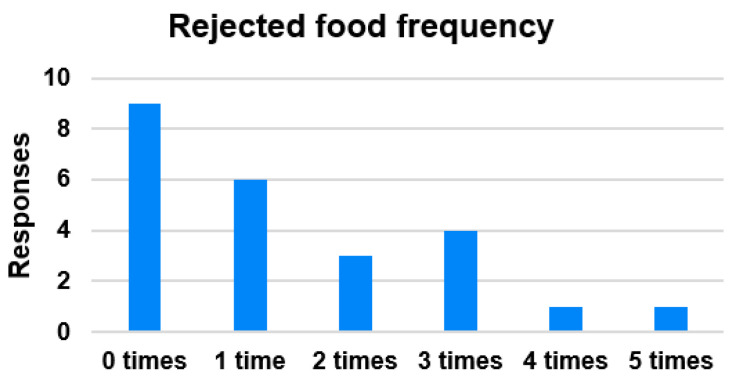
How frequently children are unsatisfied with a lunch.

Investigating how frequently children are not satisfied with school food (Figure 5), the survey showed two peaks, which can relate to two groups: “satisfied children” and “unsatisfied children”. Of course, the sample population must be increased; however, this principle can be applied to generate the frequency of hated food based on selected assumptions: (a) 0(0.5) days for satisfied children; (b) 3(0.5) days—for unsatisfied children. Of the total children, 27% were unsatisfied with school food for more than one day.

**Figure 6:**
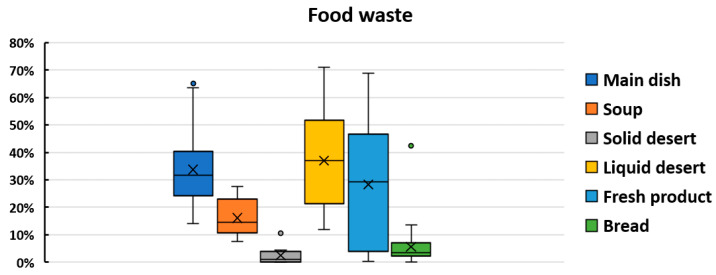
Observed food waste grouped by simulation category.

The observations at the schools showed that the largest food waste, 37%, related to liquid desserts (sweet tea, juice and lemon water); however, the total plate waste was 27.63%, and 23.28%, excluding the liquid dessert. All the categories are depicted in Figure 6. These distributions of food waste amount can be considered for rejected food: *M^w^* = *norm*(0.30, 0.05), *S^w^* = *norm*(0.15, 0.03), *Sd^w^* = 0.02, *Ld^w^* = *norm*(0.35, 0.07), *Fp^w^* = *norm*(0.3, 0.10), *B^w^* = 0.05 (*Python*).

### 3.3. The Monte Carlo Model

The time unit of the simulation model is a week. Each week the menu is presented by a set of package menus $\vec{P}=\left({\vec{p}}_{mon},\;{\vec{p}}_{tue},\;{\vec{p}}_{wed},\;{\vec{p}}_{thu},\;{\vec{p}}_{fri}\right)$, where each package menu (w) is described by Equation (1). Each menu *w* is generated considering the scheme depicted in Figure 2 and the distributions described in Table 1, where each type of menu has its own probability: (a) the main dish and a soup—65%; (b) the main dish and a dessert—30%; (c) a soup and a dessert—5%. Bread is added with a probability of 50%. Fresh products and milk are added with a probability of 33% for each; however, the type of dessert (solid, liquid or both) is selected with a uniform probability (33%). The related pseudocode is depicted in Table 6.

The plate waste is a result of two agents’ interaction: a week menu and children ($L=\langle \vec{P};C\;\rangle )$. Each child represents a set of three parameters $c=\left\{v;ef;\vec{N^{\prime }}\right\}$ capturing an eating rate, external food and the hated menu vector, which contains logical values *‘true’* or *‘false’* ($n^{\prime }=\left\{0;1\right\}$). A child generation pseudocode is depicted in Table 7. The plate waste calculation is completed in three stages: (1) competitive food impact (${\vec{w}}_{ef}^{\prime }$); (2) rejected food impact (${\vec{w}}_{h}^{\prime }$); (3) insufficient time impact (${\vec{w}}_{t}^{\prime }$), if a child decides (*d*) to stop lunch or come late to a classroom.

The core logic of the Monte Carlo model relates to plate waste calculation. It was decided to represent a lunch portion as Equation (10):

(10) $$p=e\left(t\right)+w_{t}^{\prime }\left(t\right)+w_{h}^{\prime }+w_{ef}^{\prime },$$

where $w_{t}^{\prime }$—plate waste (g) impacted by insufficient time, $w_{h}^{\prime }$—impacted by rejected food, $w_{ef}^{\prime }$—impacted by competitive food. If e(t) and $w_{t}^{\prime }\left(t\right)$ depend on time, $w_{h}^{\prime }$ and $w_{ef}^{\prime }$ Produces constant plate waste; however, the rejected food amount depends on the competitive food impact because children are already overfilled (Equation (11)).

(11) $$C=w_{h}^{\prime }\left(w_{ef}^{\prime }\right)+w_{ef}^{\prime }.$$

Mathematically, the rejected food can be calculated using Equation (12):

(12) $$\{\;\begin{array}{c} w_{h}^{\prime }=rand-w_{ef}^{\prime }\\ w_{h}^{\prime }\geq 0 \end{array}$$

The related pseudocodes are depicted in Table 8 and Table 9.

**Figure 7:**
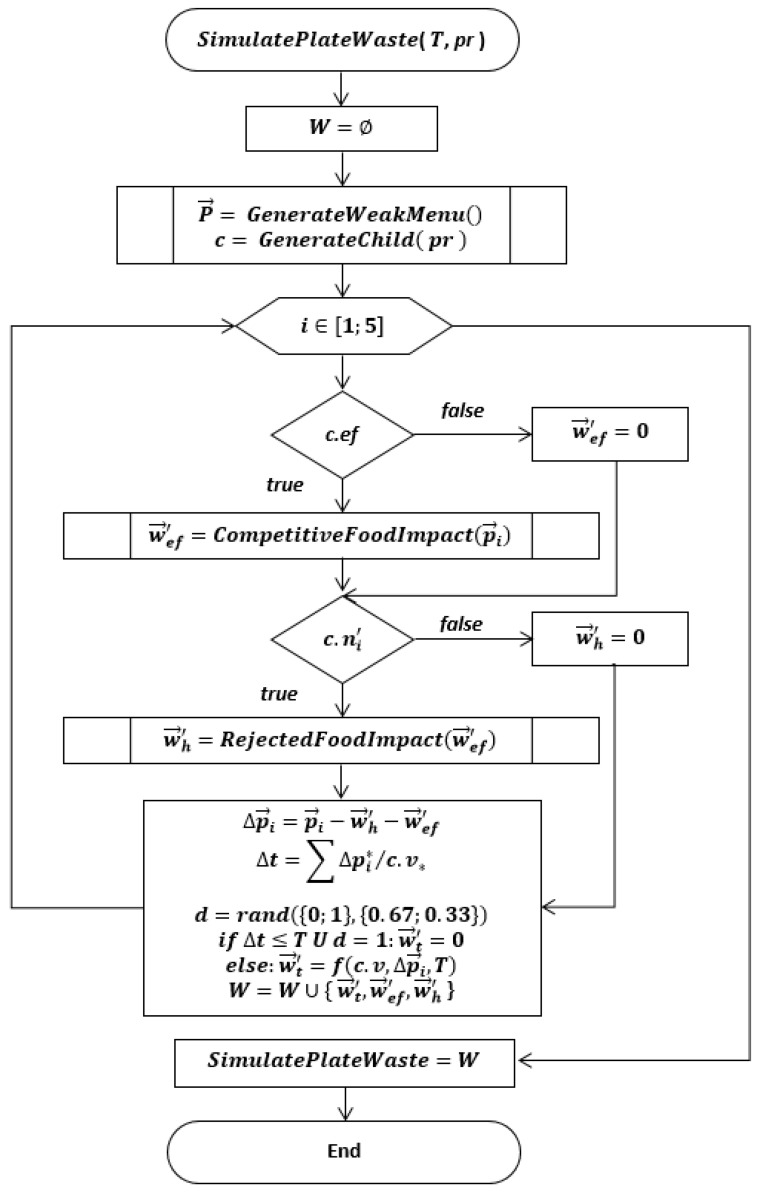
Flowchart of plate waste simulation.

The results in the form of a flowchart for plate waste simulation are depicted in Figure 7. The simulation was repeated 10,000 times to investigate the impacts of parameters.

### 3.4. Quality Control

The root mean square error (*RMSE*) was applied to measure the forecasting error of the developed simulator. Some schools had different lunch lengths for different classes; therefore, the forecasting result was compared with observed plate waste per week, considering the lunch duration (see Equation (13)).

(13) $$RMSE=\sqrt{\frac{\sum {\left(w_{t}-w_{s,\;t}^{\prime }\right)}^{2}}{N}},$$

where N—number of observations, t—lunch length corrected considering the walking time (min), s—school, $w_{t}$—forecasted plate waste per week (g/week), $w_{s,\;t}^{\prime }$—observed plate waste per week for school s, for a child group restricted by lunch duration t.

Additionally, a mean average percentage error was applied (see Equation (14)):

(14) $$MAPE=\frac{100\%}{N}\sum_{s,t}^{N}\frac{\left(w_{t}-w_{s,\;t}^{\prime }\right)}{w_{s,\;t}^{\prime }}.$$

Next, assumptions were applied for the simulation based on the previous analysis: external food probability—0.3, the probability of children unsatisfied with the school meal—0.27 and the compromise eating rate—35(5) g/min.

## 4. Experiment Results

**Figure 8:**
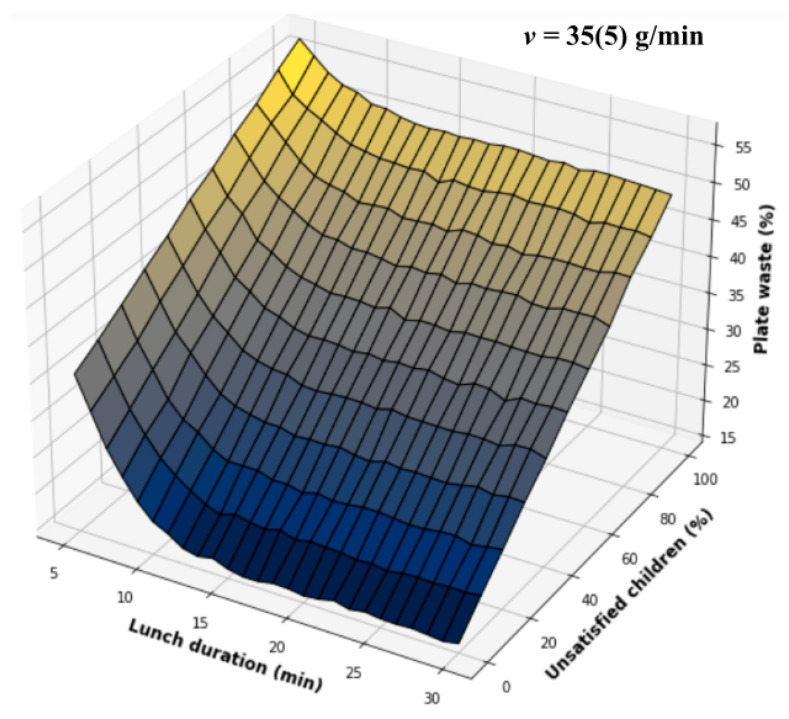
Simulation results.

**Figure 9:**
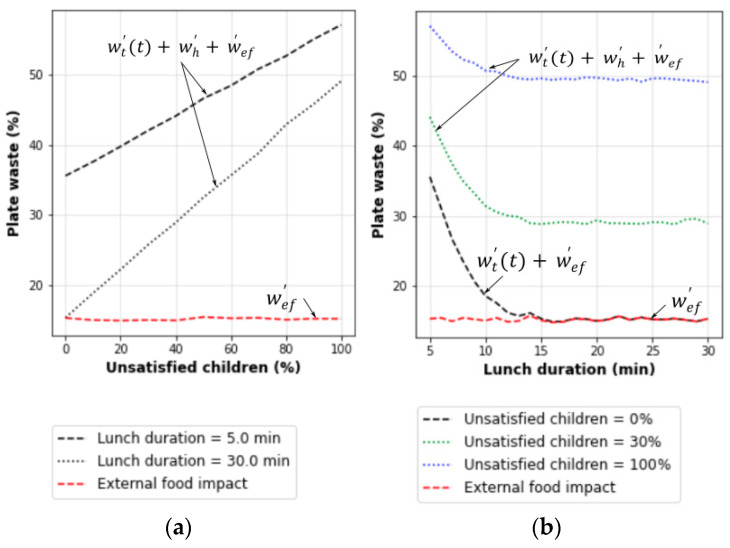
Tomograms of the simulation results: (**a**) depict the impact of rejected food on plate waste; (**b**) depict the impact of lunch duration on plate waste.

**Figure 10:**
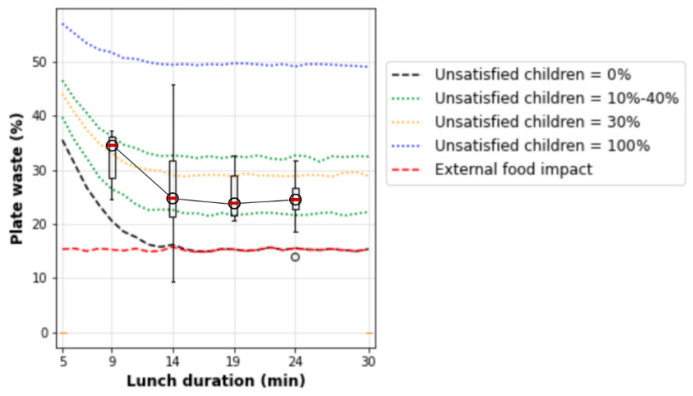
Simulation results intersected with the observed plate waste per week per class, where the boxplots are the observed plate waste.

The simulation model has two input parameters (see Figure 7): lunch duration and a percentage of children unsatisfied with school food. The results of simulations are depicted in Figure 8; however, Figure 9 depicts surface tomograms to easier understand the impact of each input parameter on plate waste. Figure 10 depicts the intersection of real data with the simulated data to validate the model.

The schools had lunch durations of 15, 20, 25 and 30 min. The observed plate waste at the schools was intersected with the simulation results adjusted for walking time. The children come into the canteen with a delay of approximately up to 3 min—the same time was considered for a return path; therefore, the total delay was equal to 6 min.

The comparison of the forecasting results with the observed plate waste showed an *RMSE* equal to 0.03.

This section may be divided into subsections. It should provide a concise and precise description of the experimental results, their interpretation, as well as the experimental conclusions that can be drawn.

## 5. Discussion

In spite of the fact that many parameters of the Monte Carlo model were based on the statistical data of the survey with only 24 responses, the quality control of the developed model showed sufficiently good results—an RMSE was equal to ±3%. The minimal and maximal absolute errors were 0.55% and 5.84% (the square errors—$2.97\times 10^{-5}$ and 3$.41\times 10^{-3}$, respectively); however, it must be considered that the *RMSE* was calculated considering the average values for all the observed schools; therefore, the error related to the prediction for a city or a region and not for each school individually. In addition, the statistical data applied for the simulator represented a summary for a city. As regards the precision, Malefors et al. [21] mention: “The past year was used for testing the models against real observations. The current business as usual scenario results in an average percentage error of 20–40%, whereas the best forecasting case has an error of around 2–3%”. The developed simulator obtained a MAPE of 10.15%.

If considering each class individually, it is possible to see large dispersion of the plate waste in Figure 10. At this moment, schools do not perform the monitoring of children’s satisfaction with school lunch. If schools survey children and collect data on unsatisfied children and the probability of competitive food for each class, it is possible to obtain precise forecasting results for each school. Additionally, the project group did not have a possibility to collect a comprehensive statistic related to path time; therefore, the samples of bounding boxes have left and right movement allowances, which increases the dispersion.

Speaking about the plate waste, Martins et al. [24] summarized the information about the plate waste, mentioning the following statistical values: 2–49% for primary school lunches in the United States of America, 23%—in Swedish school canteens and 20–29% of prepared meal in Italian schools. Figure 10 depicts comparable simulated and observed values; however, other regions must tune the developed model using local statistical data on school meals.

The developed Monte Carlo model applies two parameters for the simulation: lunch duration and the number of unsatisfied children with school meals. That does not require some complex data monitoring for plate waste forecasting. In addition, considering the relationship depicted in Figure 9, the input list of the developed model must be extended with the parameter “Competitive food probability”. It must be mentioned that the parameters—competitive food and a number of unsatisfied children—can have a strong correlation that must be studied in the future to simplify the developed model considering its practical usability for schools. Additionally, it can be time consuming for schools to complete a continuous survey of children’s preferences; therefore, it is important to consider automatization using digital tools, which can be connected to a system for menu generation to increase children’s satisfaction with school meals.

**Figure 11:**
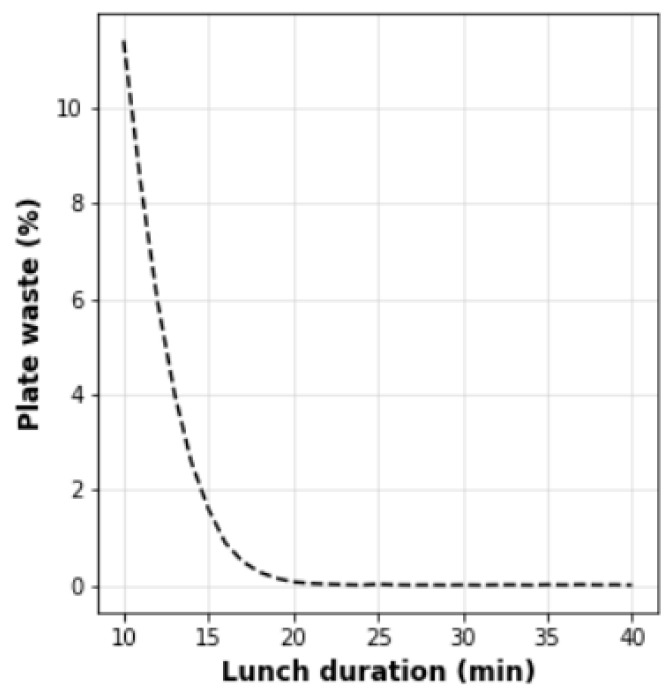
Lunch duration in the ideal conditions.

The real parameters of schools can be applied for plate waste forecasting. The mathematical models constructed in Figure 9 and Figure 10 can be used to train managers of canteens. Our simulation results precisely show that schools must primarily schedule lunch duration correctly because insufficient time provides the exponential increase in the plate waste amount (see Figure 8, Figure 9 and Figure 10). Additionally, schools must teach children about healthy food importance and monitor food preferences to minimize the rejected food amount and competitive food impact because the competitive and rejected food provides a linear increase in food waste proportional to the number of unsatisfied children (see Figure 9). In addition, it is possible to calculate the optimal lunch duration for the ideal conditions when all children are satisfied with school food and do not buy competitive food. Figure 11 depicts the plate waste amount depending on lunch duration, considering the linear model and the recalculated eating rate of 27(2) g/min mentioned by Zandian et al. [25] for girls because the eating rate of girls was lower than the eating rate of boys. Considering the obtained results, the minimal meal duration must be 20 min, excluding walking time and other external factors, which can cause food intake delay. This duration is longer than our previously proposed lunch duration (15 min) based on smaller data amount [26], which is more consistent with the durations found by Cohen et al. [32] and Hamdi et al. [33], 20 min and 17/18 min, respectively.

**Figure 12:**
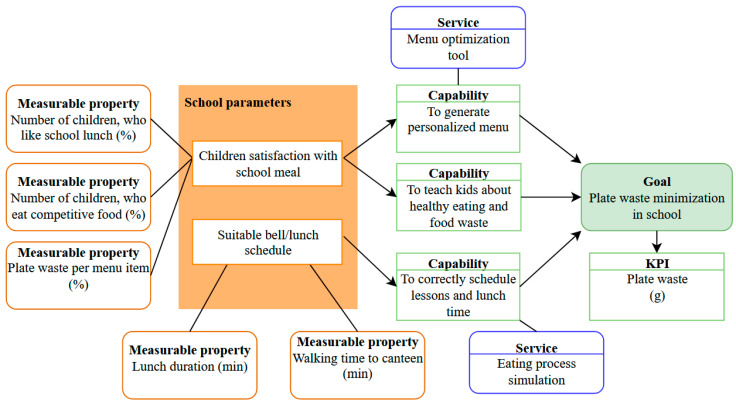
Capability model for effective decision making on food management in schools.

It is possible to construct a model for effective decision making to minimize the plate waste of schools based on the simulation results and the developed simulator itself. The capability model for effective decision making on food management in schools is depicted in Figure 12, where the developed simulator is one of the services, which can be applied to correctly schedule lessons and lunchtime and to train school managers; however, a menu optimization tool must be developed to personalize a menu for children to reduce the number of kids who are not satisfied with school meals and eat competitive food. In addition, measurable properties of children’s satisfaction with school meals can be monitored by some IoT solution or data can be collected through mobile application/surveys.

## 6. Conclusions

The simulator developed by using the Monte Carlo method showed an *RMSE* equal to ±3% and a MAPE of 10.15%, which represents the best forecasting case mentioned by Malefors et al. [21]; however, many parameters of the Monte Carlo model applied in the experiment were based on 24 responses. The practical usage of the developed simulator requires a larger sample, and the target population must be the educatees of the school where the forecasting tool (simulator) will be applied. An *RMSE* was calculated for all schools; however, the absolute error for each school obtained a maximal value of 5.84%, which can be reduced using the data on the target school. At the same time, the pseudocode for child generation (Table 7) is based on the distribution depicted in Figure 5, which showed two peaks, but it can have an exponential distribution; however, it is not very important because each school can have its own specific distribution. It is more important to mention the limitation of the developed simulator—data updating—therefore, the developed simulator must be supported by the periodical surveys completed by schools; additionally, these surveys can tune the simulator for another target region or school. Further, the developed simulator can be applied as a decision-making tool and a training tool. 

The Monte Carlo method identified that schools must primarily correctly schedule lunch duration because insufficient time leads to an exponential increase in plate waste amount. In the ideal conditions, the simulator showed that the minimal meal duration must be 20 min, excluding walking time. After that, schools must teach children about healthy food importance and negative food waste impacts and monitor food preferences to minimize the rejected food amount and decrease the competitive food probability. The continuous monitoring of children’s preferences and their surveys are time-consuming processes that could be automated by digital tools connected to a menu generation system.

The developed capability model for effective decision making on food management in schools (Figure 12) considers the plate waste only. It does not consider the quality of the meal intake; however, the system could be extended in the future if a computer vision system is developed to measure plate waste components, as well as an ontology, which matches a product with macro and micronutrients. As a result, it will be possible to recalculate the plate waste into insufficient meal intake expressed in macro and micronutrients. However, if a school meal is developed considering nutrition recommendations for a healthy lifestyle, it is a challenge to join these data with data on a competitive food; it can be achieved if children have a personalized health monitoring system, which can import school data and information from their private monitoring systems.

## Figures and Tables

**Table 1:** Simulation parameters for meal portion generation.

Meal Component	Weight (g)	Distribution	Python
Main dish (*M*)	[150; 430]	gumbel	np.random.gumbel (225, 37.5)
Soup (*S*)	[150; 300]	normal	np.random.normal (225, 37.5)
Solid dessert (*Sd*)	[50; 100]	normal	np.random.normal (75, 12.5)
Liquid dessert (*Ld*)	[150; 250]	normal	np.random.normal (200, 25)
Bread (*B*)	[20; 35]	exponential	np.random.exponential (1.2) + 20
Fresh product (*Fp*)	[50; 100]	normal	np.random.normal (75, 12.5)
Milk (*Mk*)	200	const	200
Main dish & soup (MS)	[150; 250] + [150; 200]	normal × 2	np.random.normal (200, 25) np.random.normal (175, 12.5)
Solid & liquid dessert (*D*)	[15; 50] + [100; 200]	normal × 2	np.random.normal (32.5, 8.75) np.random.normal (150, 25)

**Table 2:** Menus of schools in the observation week.

Day	*m* (g)	*s* (g)	*sd* (g)	*ld* (g)	*b* (g)	*fp* (g)	*mk* (g)	*p* (g)
Mon	300	0	40	210	25	0	0	575
Tue	205	0	180	200	25	25	0	635
Wed	230	255	0	200	25	30	0	740
Thu	230	0	180	200	25	30	0	665
Fri	240	125	0	200	25	0	0	590

*m*—a main dish, *s*—a soup, *sd*—a solid dessert, *ld*—a liquid dessert, *b*—bread, *fp*—a fresh product, *mk*—milk, *p*—a portion size.

**Table 3:** Survey answers about competitive food impacts on the package menu.

Answers	Impact of the School Optional Menu	Impact of Competitive Food from Outside the School
Eat 0–24%	22.2%	25.0%
Eat 25–49%	11.1%	25.0%
Eat 50–74%	33.3%	25.0%
Eat 75–100%	33.3%	25.0%

**Table 4:** Survey answers about the sources of competitive food.

Answers	Home Food	School Optional Menu	Outside the School	Probability of Competitive Food
Yes	12.5%	33.3%	16.7%	21.0%
No	75.0%	45.8%	54.2%	58.0%
Sometimes	12.5%	20.8%	29.2%	21.0%

**Table 5:** Children’s food preferences.

Answers	Do Not Eat Soup	Do Not Eat the Main Dish	Do Not Drink Sweet Drinks
Yes	50%	8%	0%
No	50%	92%	100%

**Table 6:** Pseudocode for week menu generation.

Attributes	Pseudocode
k—type of menu; *MS*—distribution “main dish & soup”; MD—distribution “main dish & dessert”; SD—distribution “soup & dessert”; m—main dish (g); s—soup (g).	Generate the main part:m=s=0k←rand({MS, MD, SD}, {0.65, 0.30, 0.05})if k=MD: m∈MDelif k=SD: s ∈SDelse: {m, s}∈MS
d—type of dessert; Sd—distribution “solid dessert”; Ld—distribution “liquid dessert”; D—distribution “solid & liquid desserts”; sd—solid dessert (g); *ld*—liquid dessert (g).	Generate a dessert:sd=ld=0d←rand( {Sd, Ld,D} )if d=Sd: sd∈Sdelif d=Ld: ld∈Sdelse: {sd, ld}∈D
o—type of optional part; B—distribution “bread”; Fp—distribution “fresh products”; Mk—distribution “milk”; fp—fresh products (g); *mk*—milk (g).	Generate an optional part:b=fp=0if rand({0;1}): b∈Bo←rand( {Fp, Mk, ∅} )if o=Fp: fp∈Fpelif o=Mk: mk∈Mk
$\vec{p}$—meal portion.	$\mathrm{return}:\;\vec{p}=\left\{\;m,\;s,\;sd,\;ld,\;b,\;fp\;\right\}$

Distributions of categories are provided in Table 1.

**Table 7:** Pseudocode for child generation.

Attributes	Pseudocode
pr—probability that a child is unsatisfied with school food; $v_{s}—\mathrm{eating}\;\mathrm{rate}\;\mathrm{of}\;\mathrm{solid}\;\mathrm{food}\;(g/\min);$ $v_{l}—\mathrm{eating}\;\mathrm{rate}\;\mathrm{of}\;\mathrm{liquid}\;\mathrm{food}\;(g/\min);$ ef—user of competitive food; *u*—child unsatisfied with school food.	Generate a child (pr): $v_{s}=norm\left(\;35,\;5\;\right)\;v_{l}=2\cdot v_{s}\;ef=rand\left(\;\left\{0;1\right\},\;\left\{0.7;0.3\right\}\;\right)\;u=rand\left(\;\left\{0;1\right\},\;\left\{1-pr;pr\right\}\;\right)$
${\vec{N}}^{\prime }$—days of rejected food.	$\mathrm{Generate}\;\mathrm{days}\;\mathrm{of}\;\mathrm{rejected}\;\mathrm{food}(u):\;if\;u=1:h=norm\left(\;3,\;0.5\;\right),\;0\leq h\leq 5\;else:\;h=norm\left(\;0,\;0.5\;\right),\;0\leq h\leq 5\;{\vec{N}}^{\prime }=\{\;n^{\prime }|\;N^{\prime }\left(1\right)=h\;\&\;\;N^{\prime }\left(0\right)=5-h\;\}\;$
c—child.	$\mathrm{return}:\;c=\left\{\;\vec{v},\;ef,\;{\vec{N}}^{\prime }\;\right\}$

**Table 8:** Pseudocode for competitive food impact calculation.

Attributes	Pseudocode
$\vec{p}—\mathrm{meal}\;\mathrm{portion};\;k—\mathrm{average}\;\mathrm{impact}\;\mathrm{of}\;\mathrm{competitive}\;\mathrm{food};$ ${\vec{w}}_{ef}^{\prime }$—plate waste due to competitive food.	$\mathrm{Calculate}\;\mathrm{competitive}\;\mathrm{food}\;\mathrm{impact}\;(\vec{p}):$ $k=rand\left(\;\left\{0.12,\;0.37,\;0.62,\;0.87\right\}\;\right)\;{\vec{w}}_{ef}^{\prime }=\vec{p}\cdot \;norm\left(\;k,\;0.06\;\right)$
	$\mathrm{return}:\;{\vec{w}}_{ef}^{\prime }=\left\{\;w_{ef}^{m},\;\;w_{ef}^{s},\;\;w_{ef}^{sd},\;\;w_{ef}^{ld},\;\;w_{ef}^{fp},\;\;w_{ef}^{b}\;\right\}$

**Table 9:** Pseudocode for rejected food impact calculation.

Attributes	Pseudocode
${\vec{w}}_{h}^{\prime }$—plate waste due to rejected food; $\left\{M^{w},\;S^{w},\;Sd^{w},\;Ld^{w},\;Fp^{w},\;B^{w}\right\}$ waste distributions by food categories; $h_{s}$ child does not eat soup; $h_{m}$—child does not eat the main dish.	$\mathrm{Calculate}\;\mathrm{rejected}\;\mathrm{food}\;({\vec{w}}_{ef}^{\prime }):$ ${\vec{w}}_{h}^{\prime }=\vec{p}\cdot \left(M^{w},\;S^{w},\;Sd^{w},\;Ld^{w},\;Fp^{w},\;B^{w}\right)\;h_{s}=rand\left(\;\left\{0;1\right\}\;\right)\;if\;h_{s}=1:\;w_{h}^{s}=p^{s}\;h_{m}=rand\left(\;\left\{0;1\right\}\;,\;\left\{0.08,\;0.92\right\}\;\right)\;if\;h_{m}=1:\;w_{h}^{m}=p^{m}\;{\vec{w}}_{h}^{\prime }=p-{\vec{w}}_{ef}^{\prime },\;\mathrm{where}\;w_{h}^{i}\geq 0$
	$\mathrm{return}:\;{\vec{w}}_{h}^{\prime }=\left\{\;w_{h}^{m},\;\;w_{h}^{s},\;\;w_{h}^{sd},\;\;w_{h}^{ld},\;\;w_{h}^{fp},\;\;w_{h}^{b}\;\right\}$

## Data Availability

All datasets generated during the current study are available from the corresponding author upon reasonable request.
